# Seasonal Distribution and Diversity of Non-Insect Arthropods in Arid Ecosystems: A Case Study from the King Abdulaziz Royal Reserve, Kingdom Saudi Arabia

**DOI:** 10.3390/biology13121082

**Published:** 2024-12-22

**Authors:** Taghreed A. Alsaleem, Moutaman Ali Kehail, Abdulrahaman S. Alzahrani, Turki Alsaleem, Areej H. Alkhalifa, Abdulaziz M. Alqahtani, Mohammed H. Altalhi, Hussein H. Alkhamis, Abdullah M. Alowaifeer, Abdulwahed Fahad Alrefaei

**Affiliations:** 1 The King Abdulaziz Royal Reserve (KARR), Riyadh 12213, Saudi Arabia; t.aalsaleem@karnr.gov.sa (T.A.A.); a.alzahrani@karnr.gov.sa (A.S.A.); t.alsaleem@karnr.gov.sa (T.A.); t.abdulaziz@karnr.gov.sa (A.M.A.); t.mohammed@karnr.gov.sa (M.H.A.); 2 Green Sustainability Company for Environmental Services (GSCES), Riyadh 13326, Saudi Arabia; motaman@gsces.sa (M.A.K.); hussein@gsces.sa (H.H.A.); 3 Department of Biology, College of Science, Princess Nourah bint Abdulrahman University, Riyadh 11671, Saudi Arabia; ahalkhalifa@pnu.edu.sa; 4 Department of Zoology, College of Science, King Saud University, P.O. Box 2455, Riyadh 11451, Saudi Arabia

**Keywords:** biodiversity, arachnids, crustaceans, myriapods, KARR, Saudi Arabia

## Abstract

The biodiversity of invertebrate animals is largely affected by climatic changes. This study evaluates the seasonal abundance and diversity of non-insect arthropods in the King Abdulaziz Royal Reserve (KARR), Saudi Arabia, over four collection periods (summer, autumn, winter, and spring) during 2023. Sampling was conducted across multiple sites in the reserve using both active (manual collection and active surveying for the diurnal species) and passive (pitfall traps and malaise traps for the nocturnal species) methods. A total of 586 non-insect arthropod specimens were collected, representing four classes: Arachnida, Chilopoda, Branchiopoda, and Malacostraca. The results show that the most abundant species was the jumping spider *Plexippus paykulli*, which dominated collections across two seasons, with a peak abundance of 50.7% in late summer. Seasonal variations in non-insect arthropod diversity were observed, with a lower diversity recorded during January–March (4 species, and this may be attributed to this period revealing the lowest temperature reading recorded during the study period) and higher diversity in August–September (end of summer) and October–November (mid of autumn), with 14 species. Scorpions, particularly species from the families Buthidae and Scorpionidae, were common during the summer months, while solifuges and centipedes showed sporadic occurrences across seasons. These findings align with the results for arthropod distribution in arid regions, with temperature and resource availability as key drivers of biodiversity in desert environments because of their direct effects on the biochemical processes of these creatures. This study contributes valuable baseline data on the non-insect arthropod fauna of the KARR. The insights gained from this study can aid in conservation efforts and provide a foundation for further research on non-insect arthropod ecology in arid landscapes.

## Simple Summary

A well-designed conservation strategy for natural reserves cannot be accomplished without providing the necessary information regarding the abundance and density of animal fauna in respect to seasonal variation and the floral components. Using active and passive survey methods in some sites representing all of the ecosystems in the King Abdulaziz Royal Reserve (KARR), Saudi Arabia, the first biodiversity survey was conducted during winter (January–February), spring (May), summer (August–September), and autumn (October–November) of the year 2023. The results of this survey can be considered as baseline data for the KARR (to be compared spatiotemporally with further surveys), and it reflected the abundance and density (in term of Simpson’s index) of some species of scorpions, spiders, centipedes, malacostraca, and branchiopods, among other groups of fauna and flora. The obtained baseline data will help to monitor the threshold of any threats. This study strongly recommends continuing routine seasonal surveys (to support the baseline data), in parallel with other ecological studies aimed to assess the interactions between the biotic and abiotic components of the KARR ecosystem.

## 1. Introduction

Land-use transformation and habitat degradation are key drivers of global biodiversity loss, particularly in vulnerable ecosystems such as deserts [1]. In Saudi Arabia, diverse ecosystems such as arid deserts, mountains, and savannas are present, although the country lacks permanent rivers [2]. The region faces extreme temperatures, ranging from 45–54 °C in summer to as low as −12 °C in winter, accompanied by an annual average rainfall of only 300 mm [3]. The vegetation of Saudi Arabia, specifically in the desert and scarcely vegetated areas, is composed of dwarf and xeromorphic shrubs. Annual plants usually comprises 60% of the vegetation cover, while perennial species produce new shoots during rainy season. Sandy deserts dominate, with shrubs and shrublets, in addition to some grasses [4]. These harsh environmental conditions create unique challenges for wildlife, leading to adaptations in arthropods such as scorpions [5] and camel spiders (Solifugae) [6], which exhibit behaviors like burrowing and nocturnal activity to avoid heat and conserve moisture, similar to the behaviors of species found in other desert regions like the Sahara.

Within this context, the King Abdulaziz Royal Reserve (KARR) serves as a vital haven for biodiversity, particularly for invertebrate species. Among these species, the Tenebrionidae family (darkling beetles) is well adapted to arid environments and plays a crucial role in the reserve’s detritivore community. Additionally, the painted lady butterfly (*Vanessa cardui* Linnaeus, 1758) serves as an important prey source for various predator species, while the ant species *Camponotus* functions both as a predator and a key agent for seed distribution.

The dynamic alterations in environmental conditions (e.g., climatic factors) have a notable effect on insect fertility, feeding, survival, and dispersal rates, in addition to their population dynamics, which in turn reflected on their abundance, distribution, and life cycles. Recent details exploring and explaining the complex relationships between climatic factors and insect ecology have been reported [7]. 

Invertebrates play significant roles in pollinating flowering plants, feeding predators and altering soil structure and fertility [8], and cycling nutrients [9]. Arthropods, in particular, are potential indicators of subtle habitat changes because they respond to environmental shifts at a finer scale than do larger organisms and require smaller habitat patches for survival [10]. They are the most abundant consumers in African savannas and in some instances, exhibit a greater biomass than do vertebrates [11].

Arthropods are considered the largest animal phylum, and they have adapted to inhabit marine, freshwater, soil, plant, animal body, and air ecosystems. This phylum involves four major groups: chelicerates (e.g., spiders and scorpions); myriapods (e.g., centipedes); crustaceans (e.g., Triops and woodlouse); and insects [12,13]. From over 50,000 spider species identified worldwide, some species were recorded in Saudi Arabia, among which were the brown recluse (*Loxoscekes reclusa* Gertsch and Mulaik, 1940); jumping spider (*Plexippus paykulli* Audoulin, 1826) and (*Hasarius adansoni* Audoulin, 1826); sand-dwelling Huntsman spider (*Cerbalus aravaensis* Levy, 2007); dwarf weaver (*Prinerigone vagans* Audoulin, 1826); camel spider (*Galeodes araneoides* Pallas, 1772); wolf spider (Lycosidae); cellar spider (Pholcidae); harvestmen (Opiliones); and common house spider (*Parasteatoda tepidariorum* C. L. Koch, 1841) [14].

Centipedes (Myriapoda: Chilopods) are usually abundant in agricultural soil and forest systems. Most of them feed on various invertebrates (e.g., insects and earthworms), and sometimes small vertebrates [15]. Due to their ability to feed on alternative prey, centipedes can live in various ecosystems, and they are termed as generalist predators [16]. Triops (Crustacea: Notostraca), which are recognized by their horseshoe dorsal carapace, are adapted to live in temporal freshwater bodies [17].

Scorpions (Arachnida: Sorpiones) are primarily desert-dwelling (sand-loving) arthropods that are abundant worldwide, except in Antarctica [18]. It exhibits great diversity in tropical and subtropical regions and decreases toward the cold poles. It can be found in some mountains (rock-loving) and caves, but it does not prefer dense and cold forest ecosystems such as tundra [19]. Generally, scorpions are classified as endangered invertebrates [20].

Arachnids, insects, and centipedes usually interact with each other in the same manner as with other arthropods and plants in many ways. To increase our knowledge of these relationships, field monitoring would be very useful [21]. The biodiversity in Saudi Arabia is very rich by virtue of the availability of a wide range of ecosystems. Wild and domesticated vertebrates and invertebrates have been identified and documented [22]. This study aims to set a baseline for the biodiversity of non-insect arthropods within the study area during four seasons from January to November 2023, focusing on their abundance, distribution, and density in order to develop effective management strategies for conservation.

## 2. Materials and Methods

### 2.1. Study Area

**Figure 1:**
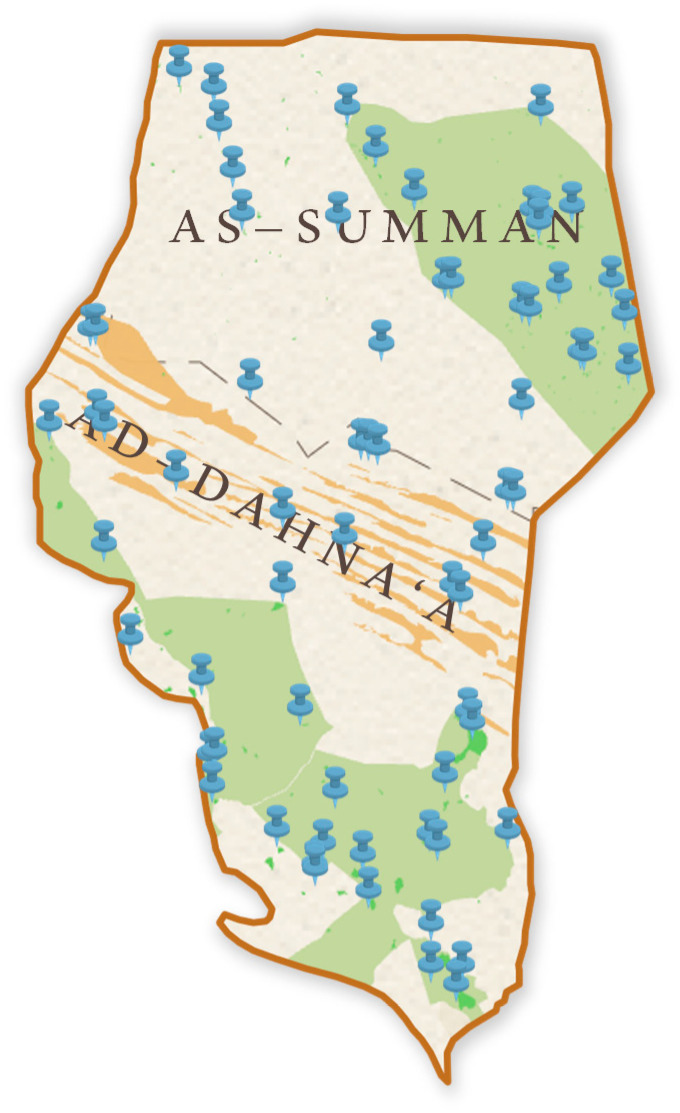
The map of the study area (KARR).

The KARR is located between coordinates E 25.512741–46.90385 and N 25.47249–27.591117, spanning two of Saudi Arabia’s thirteen administrative provinces, Riyadh Province and the Eastern Province, with a total area of 28.345 km^2^. The southern and central parts of the reserve are situated in the northeastern Riyadh Province, covering an area of 15.892 km^2^. The northern part (As-Summan) covers 12.436 km^2^. The figure below provides the revised coordinates of the KARR after boundary adjustments (Figure 1).

### 2.2. Study Periods and Topography

**Figure 2:**
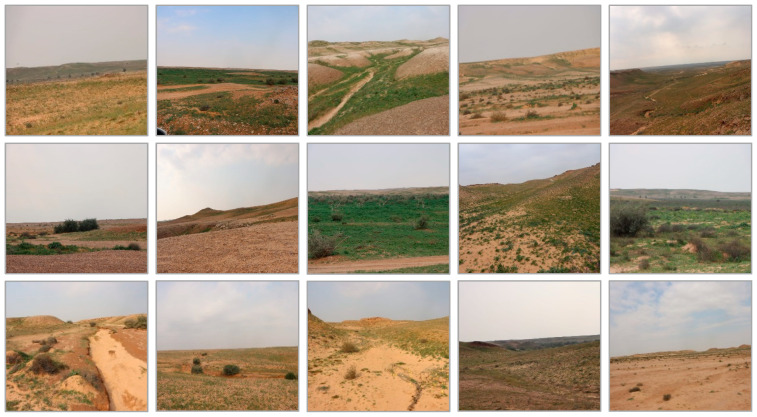
Study area topography and trap site distribution.

Due to its location, the KARR experiences a semi-arid to arid desert climate, characterized by hot days, cold nights, and minimal annual precipitation. The central and southern parts of the KARR, including Al-Tanahat, Al-Khafs, Noura, and the Ad-Dahna’a Desert, lie within the eastern geological section of Riyadh Province, known as Ar-Raf Alarabi (the Arabian Shelf). This area occupies the eastern part of the Najd Plateau, extending from Nufud Al-Sir in the west to the Ad Dahna region in northeastern Riyadh Province (Figure 2). The southern portion of the KARR represents approximately 5.9% of the Arabian Shelf, formed over ancient geological periods.

### 2.3. Sample Collection and Identification

This study employed active sampling methods, such as the manual collection, and immediate photography for the diurnal species. The active sampling method also involved night surveying, mostly for scorpions, using a UV-light torch [23]. The study also adopted passive methods [24], which were useful for identifying nocturnal species and for the species that hide in response to movement of the team members in the search area. Passive methods included the use of pitfall traps, malaise traps, water traps, and barber traps filled with appropriate attractant solutions. Each trap was set up at 6:00 a.m. and collected after 24 h. The collected samples were transferred to labeled bottles half-filled with 70% ethanol and then transported to the laboratory for identification.

Identification relied mainly on the morphological characteristics using the taxonomic keys for non-insect arthropods (Arachnids, Malacostraca, Chilopoda, and Branchiopoda) provided by Jackman [25], Rogers et al. [26], Sureshan et al. [27], and Wojewodka-Przybyl et al. [28]. Additionally, local field guides were used to confirm the obtained identification, since these invertebrates were previously identified in Kingdom Saudi Arabia.

### 2.4. Statistical Analysis

Data collected included density (%), distribution (number and location of collection sites), and the number of identified classes, orders, families, and species. The data were presented in tables, showing the number of positive collection sites and total collected specimens. Simpson’s Diversity Index (dominance index: D) was used to quantify the biodiversity of any given species in a community in a certain area. It is usually calculated as 1-D; hence, the higher the value, the higher the diversity (richness). It was calculated following the method of AlHajri [29], which considers both the number of individual species and their relative abundance, as follows:

$$D=\frac{\sum n\left(n-1\right)}{N\left(N-1\right)}$$

where:

N = the total number of individual species in the population.

n = the number of individual species.

The compliment (1-D) value ranges from 0 (indicating no distribution) to 1.0 (indicating distribution across all sites).

## 3. Results

### 3.1. The Collection for January–March 2023

**Figure 3:**
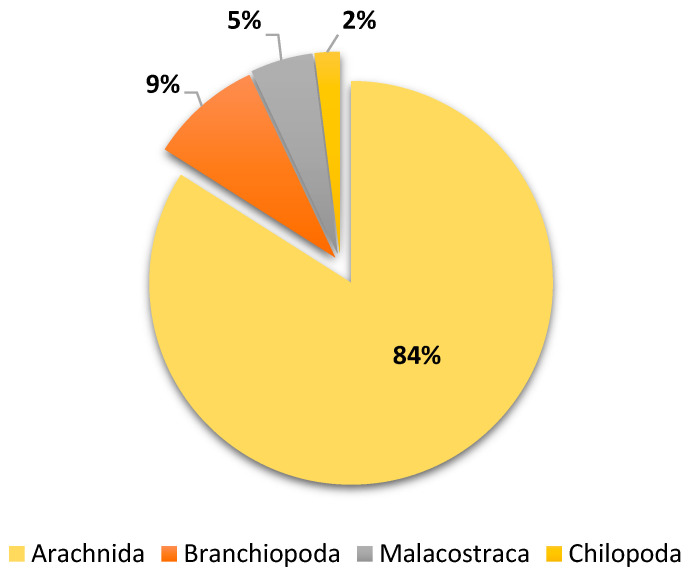
Biodiversity of non-insect arthropods trapped during January–March 2023.

Four types of non-insect arthropods were collected and identified. Spider species *P. paykulli* made up 84% of the collection, with the most common species being *Scolopendra gigantea* Linnaeus, 1758 (Chilopoda, 2%), *Triops longicaudatus* LeConte, 1846 (Branchiopoda, 9%), and *Tilos punctatus* Holmes and Gay, 1909 (Malacostraca, 5%) (Table 1 and Figure 3).

### 3.2. The Collection for May 2023

**Figure 4:**
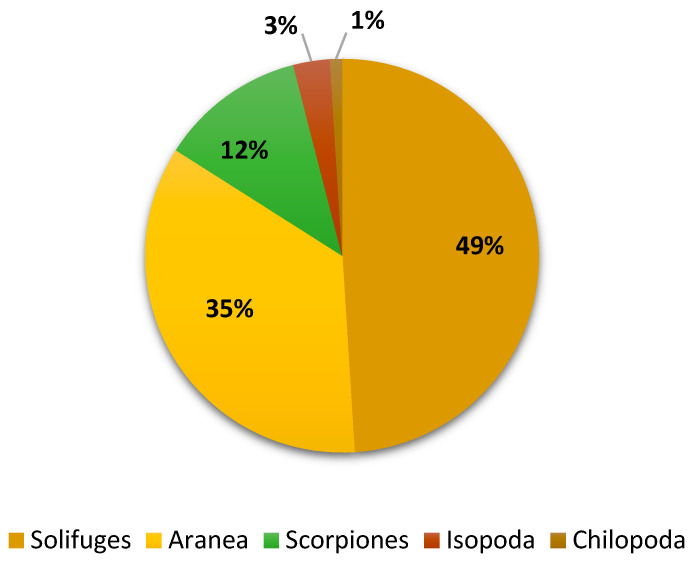
Biodiversity of non-insect arthropods trapped during May 2023.

**Figure 5:**
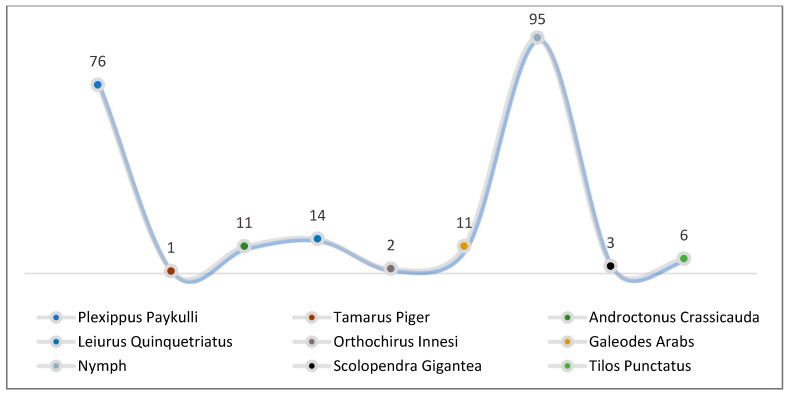
Density of each species identified during May 2023.

The May collection included three spider orders: Solifugae (49%, with both adult and nymph phases of a single species), Scorpions (12%, with three species from one family), and Araneae (35%, with two species from two families). Furthermore, one species each from the classes Malacostraca (3%) and Chilopoda (1%) was found. Along with jumping spiders (*P. paykulli*, 34.7%), Solifuge nymphs accounted for a high density (43.39%) of all specimens of non-insect arthropods (Table 2 and Figure 4). The density of each species identified is presented in (Figure 5).

### 3.3. The Collection from August–September 2023

**Figure 6:**
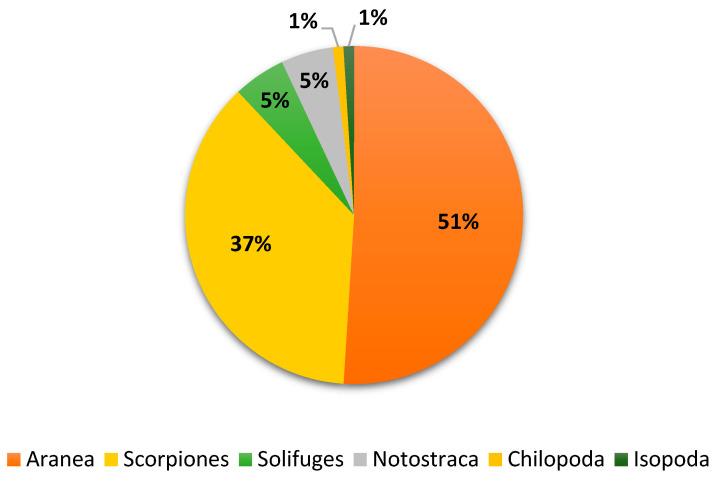
Biodiversity of non-insect arthropods trapped during August–September 2023.

**Figure 7:**
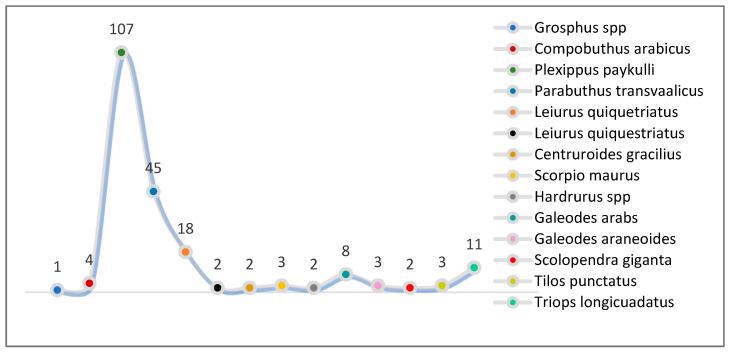
Density of each species identified during August–September 2023.

The most abundant spider orders collected during this period inlcuded Araneae (51%), Scorpions (37%), Solifugae (5%), Chilopoda (1%), Malacostraca (1%), and Branchiopoda (5%). There were five families and eleven species in these orders, including eight species of scorpions (Figure 6 and Table 3). Furthermore, Figure 7 shows the density of each of the identified species during August–September 2023.

### 3.4. The Collection from October-November 2023

**Figure 8:**
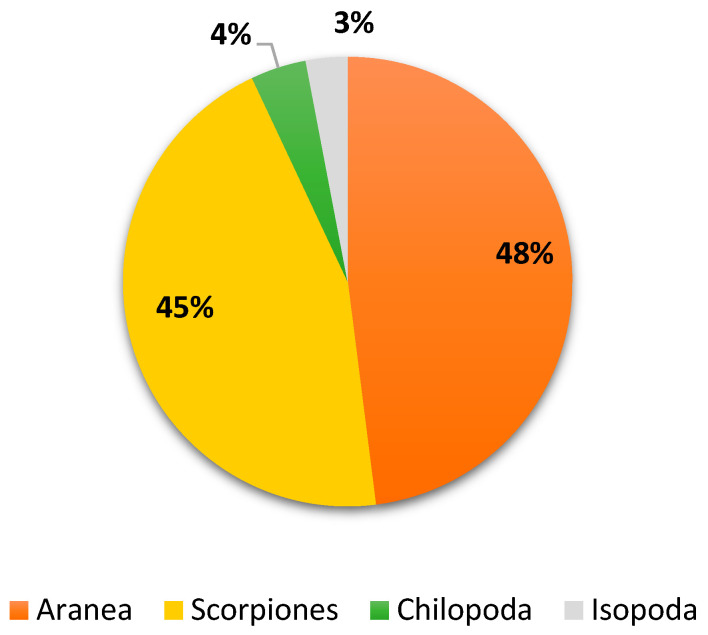
Biodiversity of non-insect arthropods trapped during October–November 2023.

**Figure 9:**
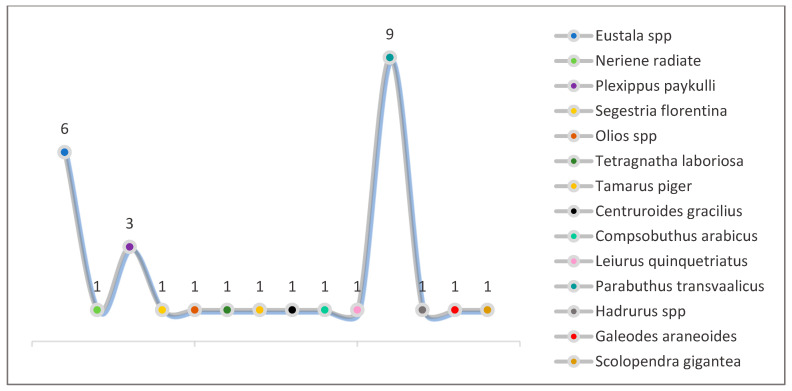
Density of each species identified during October–November 2023.

Three spider orders were present in the October–November collection: Scorpions (45%, with five species from two families), Araneae (48%, with seven species from seven families), and Solifugae (3%, with one species from one family). Additionally, one species (4%) from the class Chilopoda was found (Figure 8 and Table 4). The densities of the identified species are presented in (Figure 9).

## 4. Discussion

Studies concerned with the biodiversity of arthropods in Saudi Arabia were conducted from 2017 to 2022 [30,31,32,33,34], which also documented the influence of seasonality on arthropod populations in arid environments.

The biodiversity of similar organisms has been reported for other continents and countries. In the USA, animal biodiversity is estimated to include about 432 species of mammals, 800 species of birds, 311 species of reptiles, 295 species of amphibians, 1154 species of fishes, and more than 100,000 species of arthropods [35]. In Australia, 75% of the invertebrate group comprises insects (with more than 28,000 species of Coleoptera, 20,000 species of Lepidoptera, 14,000 species of Hymenoptera, 7700 species of Diptera, 5500 species of Hemiptera, and 2800 species of orthoptera). The arachnids include 79 species of spiders, 1000 species of mites and ticks, 150 species of pseudo-scorpions, and 120 species of scorpions [36]. In Europe, there are about 100,000 arthropod species, of which there are 500 chilopoda, 1500 diplopoda, 900 species of maxillopods, 400 species of ostracods, 1.500 species of isopods, 500 species of amphipods, 30 species of decapods, and 4100 species of spiders. Three scorpion species (*Euscorpius*, *Belisarius*, *Lurus*) are found only in the southern parts of Europe [37].

In this study, the *P. paykulli* spider was observed as the most frequently encountered species across winter (January–March) and summer (August–September), i.e., it survived and flourished at the minimum as well as at the maximum temperature rates. This spider is widely distributed in the tropical regions of Africa and Asia [38]. It generally lives on and around man-made structures, although it was also found in some rural areas [39]. Its feeding preference species includes Diptera, Hemiptera, Hymenoptera, Lepidoptera, Odonata, Orthoptera, and other spiders [40]. The abundance of *P. paykulli* spiders can be attributed to the occurrence of these prey among different seasons.

The *Eustala* spider was first described in 1895 by Simon [41]. In the KARR, this spider was recorded only during October–November 2023 within six sites, but was never recorded again. This finding reflected its sensitivity to cool (winter), hot (summer), and humid (autumn) climates within the study area. This finding significantly agreed with the work of Rodrigues et al. [42], which attributed the abundance and species richness and the composition of spiders in the subtropical areas to the seasonal variation of some abiotic factors (e.g., temperature and rainfall).

In Saudi Arabia, in addition to other spider species, about 26 species of scorpions were identified and distributed across 13 geographical regions [43].

A specific spider–plant interaction was recorded in some recent studies. The suitable microclimate for spiders is found to be strongly influenced by plant architecture (flower shape, clusters of leaves, or glandular components that provide a suitable structure for web building and hunting) rather than being randomly distributed in the vegetation, with the presence of any spider in a particular plant being dependent upon it offering shelter, access to insect prey, or a camouflage background. Same advantages were gained by the plant (e.g., protection against herbivore insects or added nutritional benefits to the plant), although some disadvantages were also noticed (e.g., consumption of pollinators) [44]. Given that plant growth is ultimately affected by the sum of the environmental factors, including the biological components, it is difficult to state a general formula for all cases; rather, the issues must be studied case-by-case through spatiotemporal, well-designed studies.

Certain spiders are known to be among the most abundant and diverse arthropods in vegetation. The jumping spider (*Plexippus* spp.: Salticidae) is considered to be an active hunter, as it pursues prey, whereas other spiders remain motionless on vegetation, waiting for prey to approach. The predation activity of spiders can affect the dynamics of the prey’s biodiversity and density, as well as the predator’s population. Spiders are considered to be excellent biological control agents against plant-pest insects, specifically in agroecosystems. Some studies have addressed the spider–plant interaction as a mutualistic relationship [44]. The question of how the morphological and structural aspects of plants can affect the spider’s composition and distribution should be verified.

It was reported that more than 60 species belonging to ten families of spiders (including Salticidae) fed directly on plant products (e.g., nectar and pollen grains in their natural habitat) [45]. It was found that the interaction between insectivorous spiders, crops, and pests produced a more resilient and healthier agroecosystem [46]. In conservation areas, the primary factor guiding management activities for maintaining and improving the state of biodiversity depends upon the protected areas (core zones) and other effective area-based conservation measures (buffer zones). Routine monitoring activities are required to evaluate both species and habitats due to the complex nature of conservation programs. Several basic questions can guide conservation area managers in implementing effective biodiversity monitoring techniques [47]. 

Invertebrate and vertebrate animals are interlocked into complicated food chains and other ecological interactions. Many groups of terrestrial arthropods (including spiders, scorpions, centipedes, ants, and beetles) include small vertebrates (e.g., reptiles, birds, and mammals) in their food preferences, especially in tropical regions. For capturing prey, these invertebrates usually possess some morphological adaptations in their mouthparts (jaws, palps, and chelicerae), in addition to producing venomous secretions [48].

Temperature, as a climatic factor, influences all biological, morphological, and behavioral processes, as well as the mating interactions, of arthropods. The success of the mating process is very important for the persistence of any arthropod population. The consequences of seasonal variation are similar to those of global warming in affecting the fitness, precopulation activity, and communication of these populations. Therefore, there is a need to quantify the biodiversity, density, and ecological impacts of temperature in term of seasons [49].

In ecology, plants and arthropods are considered to be the most diverse groups by virtue of their ability to survive in a wide range of ecosystems, from deserts to polar zones. Plants and arthropods interact with each other (pollinators, protectants, dispersers, fertilizers, nest locations, predation areas, food and energy sources, etc.) [50].

Overall, the results of this study emphasize the influence of seasonal conditions on arthropod biodiversity, with observed patterns likely driven by the availability of suitable microhabitats and food resources. This seasonal abundance variation underlines the importance of year-round monitoring to capture the full extent of biodiversity. Previously, the influence of climatic conditions on the diversity and species composition of small mammals in different natural conditions has been neglected [51,52]. The findings of this study add valuable insights regarding the composition and distribution of non-insect arthropods in Saudi Arabia, offering a reference for future studies on arthropod diversity in arid and semi-arid regions.

## 5. Conclusions

This study provides a comprehensive assessment of non-insect arthropod biodiversity in the study area across four seasonal collection periods in 2023. Our findings demonstrate significant seasonal variations in arthropod abundance and diversity, likely influenced by environmental factors such as temperature, habitat conditions, and resource availability. *P. paykulli*, a jumping spider species, seemed to be the most successful non-insect arthropod across different seasons, underscoring its adaptability to the region’s conditions. In addition, the study identified diverse spider, scorpion, and centipede species, along with notable occurrences of *T. longicuadatus* and *T. punctatus*, highlighting the ecological richness of desert habitats in Saudi Arabia.

This research adds to the existing knowledge of Saudi Arabia arthropod fauna and provides baseline data crucial for future biodiversity monitoring and conservation efforts. It also underscores the importance of continuous, year-round sampling to capture the full range of species diversity in desert ecosystems, which are particularly sensitive to environmental changes, using the standard sampling methods in the study area at certain periods during each of the four seasons. By documenting species distribution and abundance across seasons, the obtained results will set a foundation for understanding the ecological dynamics of non-insect arthropods in arid and semi-arid regions, offering insights that can support the management and preservation of desert biodiversity in Saudi Arabia.

## Figures and Tables

**Table 1:** Number of species trapped and identified during January–March 2023.

Class	Order	Family	Species	(+ve) Sites	No.	1-D
Arachnida	Araneae	Salticidae	*Plexippus paykulli*	20	107	0.709
Chilopoda	Scolopendromorpha	Scolopendridae	*Scolopendra gigantea*	2	2	1.25 × 10^−4^
Branchiopoda	Notostraca	Triopsidae	*Triops longicuadatus*	1	11	0.0069
Malacostraca	Isopoda	Tylidae	*Tilos punctatus*	2	7	0.0026
Total					**127**	

**Table 2:** Number of species trapped and identified during May 2023.

Class	Order	Family	Species	(+ve) Sites	No.	1-D
1-Arachnida	1-Araneae	1-Salticidae	*Plexippus paykulli*	21	76	0.119
		2-Thomisidae	*Tamarus piger*	1	1	0.00
	2-Scorpiones	Buthidae	1-*Androctonus crassicauda*	9	11	0.0023
			2-*Leiurus quiquetriatus*	9	14	0.0038
			3-*Orthochirus innesi*	2	2	4.19 × 10^−5^
	3-Sulifugae	Galeodidae	*Galeodes arabs*	7	11	0.0023
			Nymph	14	95	0.187
2-Chilopoda	Scolopendromorpha	Scolopendridae	*Scolopendra gigantea*	3	3	1.26 × 10^−4^
3-Malacostraca	Isopoda	Tylidae	*Tilos punctatus*	5	6	6.28 × 10^−4^
Total					**219**	

**Table 3:** Number of species trapped and identified during August–September 2023.

Class	Order	Family	Species	(+ve) Sites	No.	1-D
1-Arachnida	1-Araneae	Salticidae	*Plexippus paykulli*	18	107	0.256
	2- Scorpiones	1-Buthidae	1-*Compobuthus arabicus*	4	4	2.71 × 10^−4^
			2-*Grosphus* spp.	1	1	0.00
			3-*Parabuthus transvaalicus*	14	45	0.0447
			4-*Leiurus quiquetriatus*	3	18	0.0069
			5-*Leiurus quiquestriatus*	1	2	4.51 × 10^−5^
			6-*Centruroides gracilius*	1	2	4.51 × 10^−5^
		2-Scorpionidae	*Scorpio maurus*	1	3	1.35 × 10^−4^
		3-Caraboctonidae	*Hardrurus* spp.	1	2	4.51 × 10^−5^
	3-Sulifugae	Galeodidae	1-*Galeodes arabs*	7	8	9.48 × 10^−4^
			2-*Galeodes araneoides*	3	3	1.35 × 10^−4^
2-Chilopoda	Scolopendromorpha	Scolopendridae	*Scolopendra gigantea*	1	2	4.51 × 10^−5^
3-Malacostraca	Isopoda	Tylidae	*Tilos punctatus*	1	3	1.35 × 10^−4^
4-Branchiopoda	Notostraca	Triopsidae	*Triops longicuadatus*	1	11	0.0025
Total					**211**	

**Table 4:** Number of species trapped and identified during October–November 2023.

Class	Order	Family	Species	(+ve) Sites	No.	1-D
1-Arachnida	1-Araneae	1-Araneidae	*Eustala* spp.	6	6	0.0369
		2-Linyphiidae	*Neriene radiate*	1	1	0.00
		3-Salticidae	*Plexippus paykulli*	3	3	0.0074
		4-Segestriidae	*Segestria florentina*	2	1	0.00
		5-Sparasidae	*Olios* spp.	1	1	0.00
		6-Tetragnathidae	*Tertagnatha laboriosa*	1	1	0.00
		7-Thomisidae	*Tamarus piger*	1	1	0.00
	2-Scorpiones	1-Buthidae	1-*Centruroides gracilius*	1	1	0.00
			2-*Compobuthus arabicus*	1	1	0.00
			3-*Leiurus quiquetriatus*	1	1	0.00
			4-*Parabuthus transvaalicus*	1	1	0.00
		2-Caraboctonidae	*Hardrurus* spp.	7	9	0.0887
	3-Sulifugae	Galodidae	*Galeodes araneoides*	1	1	0.00
2-Chilopoda	Scolopendromorpha	Scolopendridae	*Scolopendra gigantea*	1	1	0.00
Total					**29**	

## Data Availability

All data analyzed are included within the article.
